# Total wrist arthrodesis with and without arthrodesis of the carpoMetacarpal joint (WAWWAM): study protocol

**DOI:** 10.1186/s12891-021-04644-4

**Published:** 2021-09-08

**Authors:** David H. Owen, Diana M. Perriman, Igor Policinski, Maurizio Damiani, Paul N. Smith, Chris J. Roberts

**Affiliations:** 1 Trauma and Orthopaedic Research Unit, Canberra Hospital, Building 6 Level 1, PO Box Woden ACT, Canberra, 2606 Australia; 2 Australian National University Medical School, Level 2 Peter Baume Building 42, Linneaus Way, Canberra, 0200 Australia

**Keywords:** Randomised control trial, Surgery, Wrist, Arthrodesis, Carpometacarpal joint, Plate

## Abstract

**Background:**

It is controversial whether or not the carpometacarpal joint (CMCJ) should be included in total wrist arthrodesis (TWA). Complications commonly occur at this site and studies examining its inclusion and exclusion are conflicting. A randomised clinical trial comparing wrist arthrodesis with CMCJ arthrodesis and spanning plate to wrist arthrodesis with CMCJ preservation and non-CMCJ spanning plate has not been performed.

**Method:**

A single centre randomised clinical trial including 120 adults with end-stage isolated wrist arthritis will be performed to compare TWA with and without the CMCJ included in the arthrodesis. The primary outcome is complications in the first post-operative year. Secondary outcomes are Disabilities of the Arm, Shoulder and Hand (DASH) score, Patient Rated Wrist Evaluation (PRWE) and grip strength measured at 1, 2 and 5 years. Late complications, return to work and satisfaction will also be recorded.

**Discussion:**

It is unknown whether the CMCJ should be included in TWA. This trial will contribute to an improved understanding of optimal management of the CMCJ in total wrist arthrodesis.

**Trial registration:**

This trial was prospectively registered with the Australia New Zealand Clinical Trials Registry with identifying number ACTRN12621000169842 on the 16th February 2021.

WHO: U1111–12626523.

ANZCTR:

ACTRN12621000169842

**Supplementary Information:**

The online version contains supplementary material available at 10.1186/s12891-021-04644-4.

## Background

Total wrist arthrodesis (TWA) is indicated for a variety of wrist conditions where motion preserving procedures are contraindicated [1]. The aim of TWA is to eliminate pain and provide stability to improve function [2]. TWA with a dorsal contoured wrist arthrodesis plate has been widely adopted [3–9]. Complications of TWA with a plate include non-union, soft tissue irritation and hardware failure. The carpometacarpal joint is a common site of complications [4, 10, 11].

Inclusion of the carpometacarpal joint in TWA is debated and complications at this site are mentioned throughout the literature [2]. Early descriptions of TWA using a plate mandated inclusion of the carpometacarpal joint. Inclusion of this joint is recommended, as persistent loading of the plate across a mobile joint may result in plate failure, and to prevent this plate removal is recommended. Arthrodesis of the CMCJ at the time of TWA is indicated for localised pain or degeneration of the joint. Some authors recommend routinely including the CMCJ in TWA, while others recommend arthrodesis for heavy manual workers.

Nagy recommended against CMCJ arthrodesis noting a high frequency of non-union and pain in patients that had hardware removed [10]. In a similar study, Berling disagreed, instead preferring to fuse the carpometacarpal joint to reduce the requirement for plate removal with a second operation [11]. Rancy compared a non-carpometacarpal spanning plate to a spanning plate with carpometacarpal joint fusion in a small retrospective series and reported similar results for both treatment groups [12]. Most recently, Hernekamp compared the Medartis APTUS© 2.5 TriLock Wrist Fusion Plate, which is a specially designed non-spanning plate that enables the carpometacarpal joint to be spared to the Depuy-Synthes© CMCJ spanning LCP wrist fusion plate without CMCJ arthrodesis, reporting similar results [4].

The advantages of preserving the CMCJ in TWA is that a small amount of motion is preserved in the hand. Seven degrees of flexion/extension, 4 degrees of radio-ulna deviation and 5 degrees of pronation-supination is present in the native third CMCJ [13], which is most commonly fused in TWA. Motion at the CMCJ improves grip strength and the ability to make a fist. Disadvantages of preservation of the CMCJ in TWA are that hypermobility and pain may develop after TWA without CMCJ arthrodesis, which may be explained by increased loading of the CMCJ though an immobile wrist. It is hypothesised that this may cause accelerated degeneration of this joint and pain after TWA.

The total wrist arthrodesis with and without arthrodesis of the carpometacarpal joint study (WAWWAM study) is a single centre, multi-surgeon triple blinded randomised trial that will examine the outcome of two different treatments of the CMCJ in TWA.

### Objectives

The aim of this study is to compare the outcomes of TWA with a CMCJ spanning plate and CMCJ arthrodesis to TWA with a non-spanning plate without CMCJ arthrodesis.

Specific aims:

- To compare perioperative and short-term (up to 12 months) complications
- To compare clinical outcomes: pain, satisfaction, return to work, function, grip strength
- To compare long-term (1–5 years) complications and CMCJ problems
- To compare patient reported outcomes: Disabilities of Arm, Shoulder, Hand (DASH) and Patient Rate Wrist Evaluation (PRWE)

## Methods/design

### Trial design

This study is a single centre randomised trial. It has a parallel group design and is stratified by surgeon. Trial admission and randomisation will occur preoperatively.

### Ethics

Approval for the conduct of this study has been received from the Australian Capital Territory Health (ACT) Services Directorate Human Ethics Committee: 2020.ETH.00207.

### Participants

**Fig. 1:**
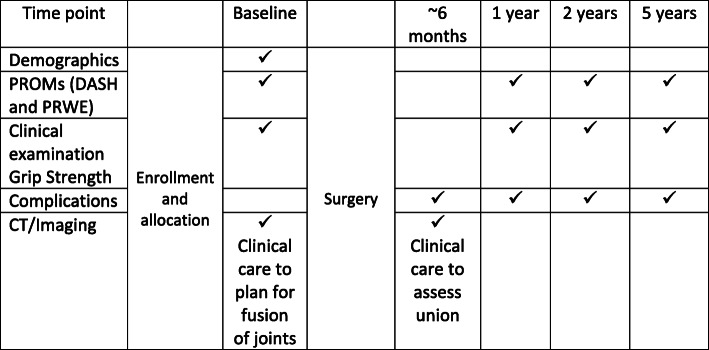
SPIRIT timeline for trial

Adults aged 18 years and older awaiting TWA will be invited to participate in this trial. Participants will be English speaking, have minimal disability of the upper limb and have non-inflammatory arthritis of the wrist. Additional eligibility criteria are specified in Table 1. The CONSORT statement is followed for the reporting of randomised trials. A participant’s journey through the trial is shown in Fig. 1.

**Table 1:** Eligibility and exclusion criteria

Eligibility criteria	
Inclusion criteria	
This study will include English speaking adults aged > 18 years who have been scheduled for total wrist arthrodesis. The conditions which are likely to be included are:	
SNAC (Scaphoid non-union advanced collapse)	
SLAC (Scapholunate ligament advanced collapse)	
Keinbock’s disease/Lunate avascular necrosis	
Preiser’s disease/Scaphoid avascular necrosis	
Wrist osteoarthritis	
Post traumatic arthritis	
Failed partial fusion	
Failed proximal row carpectomy	
Failed ligament repairs	
Exclusion criteria	
Patients will be excluded from this study if they:	
Lack ability to provide informed consent for participation (cognitive capacity or English proficiency)	
Have an inflammatory arthropathy (e.g., Rheumatoid arthritis)	
Have coexisting debilitating other upper limb disorder e.g., rotator cuff tear arthropathy with inability to raise arm above head.	
Neurological dysfunction affecting the limb of interest (CVA, plexus injury, peripheral nerve injury, spasm or contracture)	
Tumour of the wrist (giant cell or other)	
Wrist arthroplasty that will be revised to arthrodesis	
Planned to undergo or have undergone bilateral wrist arthrodesis

### Participating hospitals

The following hospitals in the Australian Capital Territory are participating in this study: Canberra Hospital, National Capital Private, Calvary John James and Calvary Bruce.

### Baseline data

A timeline for assessment is given in Fig. 1. Initial pre-operative assessment will obtain data on comorbidities, function, clinical assessment of pain at the CMCJ and radiographic assessment with x-ray and computer tomography (CT). Patient reported outcome measures including the DASH and the PRWE will also be administered.

### Randomisation and blinding

Block randomisation with stratification by surgeon will be used to ensure equal distribution of participants and size of each treatment group in the event that recruitment goals are not achieved. Eligible patients are randomised 1:1 to TWA with CMCJ arthrodesis and spanning plate or TWA without CMCJ arthrodesis with non-CMCJ spanning plate. The randomisation sequence is concealed and administered by an independent non-clinical staff member of the Trauma and Orthopaedic Research Unit at the Canberra Hospital. Letters are then allocated to participating surgeons containing the concealed treatment allocation. In this way the surgeon cannot influence the type of intervention; and the participants, clinical and research staff are blinded.

### Intervention

A fellowship trained orthopaedic surgeon with subspecialist hand surgery training will undertake the initial patient assessment and perform the surgery. All surgeons are experienced and familiar with both treatments.

Procedures are performed under general anaesthesia with antibiotic prophylaxis and the use of a tourniquet. A dorsal approach with full thickness skin flaps is used and the interval between the 3rd and 4th compartment is developed. In cases of minimal bone loss, local bone graft is used; in cases of advanced bone loss, cortico-cancellous bone graft is obtained from the ipsilateral iliac crest. If the distal radioulnar joint is arthritic, it is resected and used as bone graft. A dorsal capsulotomy is performed to expose the radiocarpal and midcarpal joint and a posterior interosseous nerve neurectomy is performed.

### Treatment a: Total wrist arthrodesis without CMCJ arthrodesis and non-bridging plate

Care is taken at this point not to damage the CMCJ or joint capsule which is identified by fine bore needle under fluoroscopy. After thorough decortication of the radiocarpal and midcarpal joints the Medartis 2.5 TriLock Wrist Fusion without arthrodesis of the carpometacarpal joint (Medartis, Hochbergerstrasse 60E, 4057 Basel Switzerland) is applied according to manufactures instructions and intra-operative imaging is obtained to ensure correct placement.

### Treatment B: Total wrist arthrodesis with CMCJ arthrodesis and bridging plate

In addition to preparing the radiocarpal and midcarpal joints, the third CMCJ is prepared for arthrodesis. The third metacarpal is marked to ensure correct rotation and a Synthes-DePuy 2.7/3.5 mm stainless steel plate (Synthes-DePuy, Eimattstrasse 34,436 Oberdorf BL Switzerland) is secured and intra-operative imaging is undertaken to confirm correct placement.

For both interventions, bone graft if used, is packed around the decorticated carpal bones. Wounds are closed in layers and interrupted nylon suture is used for skin. The wrist is then placed in a well-padded short arm volar plaster slab. Post-operative rehabilitation is standardised with immediate motion of the shoulder, elbow and fingers. At 2 weeks, participant wounds are checked, and they are supplied with a removable thermoplastic splint.

### Outcome assessment

A timeline for assessment is given in Fig. 1. Surgeons will record peri-operative complications and union on a standardised form. In each outpatient department a blinded physiotherapist will ensure post-operative assessment is completed at 1, 2 and 5 years. Patients will undergo assessment with DASH, PRWE, report pain, satisfaction and record perceived problems or complications.

### Primary outcome

The primary outcome is complication within the 12-month post-operative period. These complications are defined in Table 2. Non-union will be assessed at the radiocarpal, midcarpal and carpometacarpal joint (if arthrodesed) by CT.

**Table 2:** Complications will be defined to include the following

Wound dehiscence	
Infection: superficial (treated with antibiotics alone) and deep (requiring surgical debridement and antibiotics	
Fracture: in the perioperative period and in the follow up period	
Non-union: recorded radiocarpal, midcarpal and carpometacarpal joint and defined as absence of bridging bony trabeculae with no interval change over 3 months	
Hardware breakage: plate or screw, managed operative or non-operatively	
Tendon rupture	
Tendon irritation: requiring treatment	
Complex regional pain syndrome: defined according to Budapest criteria	
Nerve injury: operatively or not operatively managed	
Other: medical and surgical	

### Secondary outcomes

Secondary outcomes will include changes from baseline DASH and PRWE. Secondary outcomes will also include patient satisfaction, grip strength and late complications.

The DASH questionnaire is used to assess global disability of the upper limb. The questionnaire is a self-reported 30 item questionnaire that patients rate difficulty and interference with daily life on a 5-point Likert scale. The DASH is scored using the formula = ([(sum of n responses)/n] -1) where n represents the number of completed items. The score on both test ranges from 0 (no disability) to 100 (most severe disability).

The PRWE is a wrist specific questionnaire that has 15-items designed to measure wrist pain and disability in activities of daily living. The pain subscale contains 5 items each of which is further rated from 1 to 10. The function subscale contains a total of 10 items which are further divided into 2 sections: specific activities (6 items) and usual activities (4 items). Both subscales are scored out of 50, and the total score is the sum of the two scores, where 100 is the poorest possible outcome.

Grip strength will be recorded from three attempts alternating between left and right hands have using Jamar digital dynamometer in position 2.

### Radiological evaluation

Routine x-ray and pre-operative CT will be used to assess the integrity of the CMCJ. Intraoperatively fluoroscopy will be used by the surgeon to assess hardware placement, screw length and position, and alignment and rotation of the hand.

Union of the radiocarpal, midcarpal and carpometacarpal joint will be assessed by an independent specialist radiologist at 4–6 months following TWA and be defined as the presence of bridging bony trabeculae.

Non-union will be defined as the absence of bridging trabeculae, with no documented interval change over a 3-month period.

### Data collection

Data will be collected prior to surgery and in the post-operative period by the treating surgeon and at 1,2 and 5 years by blinded subspecialised hand physiotherapists, using standardised data forms (see additional files). These forms will be in locked a filing cabinet.

### Data management

Standardised data forms will be compiled by a researcher blinded to the intervention. Analysis of the data will be performed by a statistician blinded to the treatment. Digitalised data will be stored on a password protected computer.

### Sample size

This study is powered to detect a difference in the rate of complications at the CMCJ. Based on reported complications at the CMCJ in the literature and assuming alpha = 0.05, beta = 0.2 and allowing for 20% drop out, it is expected that 120 participants will be needed to discriminate between the interventions. We plan to recruit patients for this study over 3–4 years.

### Statistical analysis

All statistical analysis will be performed by a statistician blinded to treatment.

We choose a superiority study design, with the assumption that treatments are equivalent and set out to test this hypothesis. The primary outcome of complications will be analysed by proportional analysis (odds ratio). Continuous secondary outcomes will be analysed by ANOVA. This study is not powered to discriminate between treatments in terms of DASH and PRWE given the MCID (95% confidence interval) is 10 [5–15] and 14 (8–20), respectively [14].

## Discussion

The objective of this trial is to determine whether or not the CMCJ should be fused in TWA. This study will also determine the need for hardware removal and give an insight into how CMCJ is loaded following TWA by assessing the development of problems at this site.

We choose a simple study design with simple outcomes. The primary outcome is complications within 1 year of surgery. TWA are reported to unite at approximately 12 weeks and it is generally accepted that pain and function stabilise by 12 months. We therefore expect that most cases of non-union, hardware failure and soft tissue irritation will be evident by 12 months.

The secondary outcomes that we choose to examine include late complications (up to 5 years), DASH and PRWE. Nagy reported that union of the CMCJ is hard to determine, especially with hardware in situ [10]. Reigstad reported a significant number of late complications in a cohort of TWA for non-inflammatory wrist arthritis followed up for a mean of 11 years [15]. We therefore felt it important to extend the follow up period to observe undetected CMCJ non-union, indicated hardware breakage and development of CMCJ pain due to extended periods of abnormal load on the joint, as well as other complications which may be undetected within 12 months of TWA.

This randomised clinical trial will provide insight into the outcomes of TWA and may facilitate recommendation of a superior surgical method. We plan to begin recruitment of participants in early 2021.

## Supplementary Information

**Additional file 1.** Initial patient data form.

**Additional file 2.** Patient review data form.

**Additional file 3.** Surgeon review data form.

## Data Availability

Not applicable.

## Abbreviations

ANOVA: Analysis of variance
CMCJ: Carpometcarpal joint
CT: Computer tomography
DASH: Disabilities of the arm shoulder and hand
IBRA: International Bone Research Association
MCID: Minimal important difference
PRWE: Patient-rated wrist evaluation
SPIRIT: Standard Protocol Items: Recommendations for Interventional Trials
TWA: Total wrist arthrodesis
