# Supplementary material for: Total wrist arthrodesis with and without arthrodesis of the carpoMetacarpal joint (WAWWAM): study protocol
Source: BMC Musculoskelet Disord. 2021 Sep 8;22:766. doi: 10.1186/s12891-021-04644-4 (PMC8425134; doi:10.1186/s12891-021-04644-4)
Supplement: Supplementary file 1 — Additional file 1. Initial patient data form. [file 12891_2021_4644_MOESM1_ESM.docx]

**WAWWAM: Wrist arthrodesis with and without carpometacarpal joint study**

IPDF: Initial patient data form – to be collected prior to total wrist fusion

Instructions: please fill this form out. Your surgeon will help you.

| **Demographics** | |
| --- | --- |
| Name | Sex |
| Date of birth | Dominant hand |
| Occupation | Contact number |
| Address | Next of kin name and contact |
| **Medical History** | |
| Smoker? Y / N | Osteoporosis (thin bones)? Y / N |
| Ex-smoker? Y / N | Diabetes? Y / N |
| Past medical history   - Medical conditions - Previous surgery | Medications   - Drugs you currently take |
| **Wrist** | |
| Side of fusion | Previous operations on wrist to be fused |
| Reason for fusion |  |
| Date of wrist fusion |  |
| **Grip strength** use Jamar digital dynamometer in grip position 2 | |
| Right 1 | Left 1 |
| Right 2 | Left 2 |
| Right 3 | Left 3 |

Other forms attached:

1/ Patient rated wrist evaluation (PRWE)

2/ Disabilities of the Arm Shoulder and Hand (DASH)
