# Supplementary material for: Total wrist arthrodesis with and without arthrodesis of the carpoMetacarpal joint (WAWWAM): study protocol
Source: BMC Musculoskelet Disord. 2021 Sep 8;22:766. doi: 10.1186/s12891-021-04644-4 (PMC8425134; doi:10.1186/s12891-021-04644-4)
Supplement: Supplementary file 2 — Additional file 2. Patient review data form. [file 12891_2021_4644_MOESM2_ESM.docx]

**PRDF: Patient review data form – to be collected at 1 year, 2 years, 5 years after TWA**

**WAWWAM: Wrist arthrodesis with and without carpometacarpal joint study**

Please fill in this form with regard to your wrist fusion.

| **Identification** | |
| --- | --- |
| Name | Date of birth |
| **Complications – wound problems, such as additional operations, excessive pain? Please list** | |
| Satisfied? Yes/No (Please circle)  Improved? Yes/No (Please circle)  Would you have the operation again? Yes/No (Please circle) | |
| **Return to work (circle best description)**  Return to same job or occupation  Return to different job or light duties because of wrist fusion  Retired or unemployed because of wrist fusion  Retired or unemployed because of a different reason | |
| **Grip strength** use Jamar digital dynamometer in grip position 2 | |
| Right 1 | Left 1 |
| Right 2 | Left 2 |
| Right 3 | Left 3 |

Other forms attached:

1/ Patient rated wrist evaluation (PRWE)

2/ Disabilities of the Arm Shoulder and Hand (DASH)
