# Supplementary material for: Total wrist arthrodesis with and without arthrodesis of the carpoMetacarpal joint (WAWWAM): study protocol
Source: BMC Musculoskelet Disord. 2021 Sep 8;22:766. doi: 10.1186/s12891-021-04644-4 (PMC8425134; doi:10.1186/s12891-021-04644-4)
Supplement: Supplementary file 3 — Additional file 3. Surgeon review data form. [file 12891_2021_4644_MOESM3_ESM.docx]

**SRDF: Surgeon review data form – to be collected by treating surgeon**

**WAWWAM: Wrist arthrodesis with and without carpometacarpal joint study**

Instructions: please fill this form out after surgical care is complete.

| **Identification** | |
| --- | --- |
| Name | Date of birth |
| Date of TWF  Patient number (See randomisation letter) | Surgeon |
| **Wrist** | |
| Indication for wrist arthrodesis | Date of imaging demonstrating union |
| CT evidence of preop 3^rd^ CMCJ arthritis? | CT evidence of post op 3^rd^ CMCJ arthritis? |
| Previous ipsilateral wrist operations | Significant ipsilateral and contralateral upper limb problems |
| **Operation** | |
| **Operation**   - DU Excision y / n - PRC y / n   Bone graft y / n   - Type?   Other procedures such as CTD, r/o hardware | **Anaesthetic type**   - GA - Block/local infiltration |
| **Complications** | |
| Wound problems | <6 month CT wrist result   - Radiocarpal union - Midcarpal union - Carpometacarpal union |
| Complications identified during follow up |  |
| Additional wrist/hand operations subsequent to wrist fusion |  |
| **Other** | |
| List any further operations related to the wrist ie progression of arthritis required excision, chronic pain etc | |
